# Progress of Sanitary Legislation

**Published:** 1855-12

**Authors:** 


### HYGIENIC JURISPRUDENCE.

### PROGRESS OF SANITARY LEGISLATION.

*Medical Officers of Health for London.*—The chief sanitary legislative topic of the past quarter has been the election of medical officers under Sir B. Hall's new act. In order to give the municipal boards a correct idea of the kind and manner of men, who should be selected for these important duties, the Board of Health has issued a well timed document of instructions. As almost all that the Board suggests is embodied in the second article of this number (which, however, was written before the appearance, and quite independent of, the official minute) we need not copy the instructions entire. We must at the same time observe that one suggestion has by an oversight been made by the board, which to us seems simply impossible. The board recommend that in the event of one district board or vestry being unable to meet the necessary premium of a competent medical officer, two or three should unite for that purpose. This suggestion is wrong in principle; for there is no parish or district too small for the labours of one man. But more, such a course would be in opposition to the act, which states expressly, clause 132, that "every vestry and district board shall from time to time appoint one or more legally qualified medical practitioners," &c. This is definite, and cannot be modified by electors.

In the further reading of this 132nd clause, it is stated that the medical officers shall "from time to time" be appointed. The question has been asked whether this means, that the office shall not be of necessity a permanent one? To this we answer, *No*. It means that the office is permanent, but that when one officer is from any cause removed, he shall be succeeded by another.

A second question has been asked; namely, what is a legally qualified practitioner of medicine, as required by the act? To this we believe the most liberal answer is meant; namely, every man who possesses a legal qualification to practise medicine in any part of Great Britain. Any less free interpretation would be invidious, and would much cripple the electors in looking out for the most competent officers. It is absurd to defend an exclusive technical difficulty of this kind. It is equally absurd to defend the idea of selecting these officers from special grades of the profession. In medicine there ought to be grades only in talent, industry, virtue,

> "The rank is but the guinea's stamp,
> The man's the gowd for a' that."

Let the municipal councils of London look out for the man who has done most, and whose life is the dearest sacrifice to his profession and to science. Above all things, let them shun him who puts a trumpet to his mouth, to announce himself nothing but a "practical man." In nine cases out of ten they will find such an one only a practical sham, who does not know the intimate nature of the air he breathes, the water he drinks, or the physic he doses with. The parading of practical knowledge is the easiest of all cloaks for practical ignorance; and there is nothing more pitiable than to see an octogenarian child, who has despised or ignored the advance of learning in his later life, pitting his own meagre and baseless experience against modern knowledge.

We say this in a general sense; speaking truth only, but fearlessly. There are general practitioners who would fill the position of officer of health preeminently well; there are physicians who would do the same: there are some in both classes who would fill it preeminently ill—who are not made for it, nor gifted for it.

But enough of this. There are medical men on all the electing boards. Let them look out. The profession of medicine, long despised and ignored by the state, now has the opportunity to present itself to the public with influence and meaning. Let each medical elector remember this fact; and if at the day of election he, on personal grounds, gives his vote against the best candidate, let him sleep that same night on the thought that he has sold his judgment to selfishness, and his profession to dishonour.

The vestry at Paddington have taken a proper step, in suggesting the formation of a competent medical board for examining candidates for the office in their parish, and for reporting on the most competent.

"Tempora mutantur, nos et mutamur in illis." Not long ago, this sanitary science business was ignored and despised. "My practice left me," once said a sanitarian to us, "as soon as I became devoted to sanitary reform; for many of my brethren thought I was mad, and all dubbed me a theorist, and what not. So I have been poor, very poor, ever since; and that is what you will be, if you do not steer clear of the sanitary gulf, and stick to the blue bottles." The advice was good, though it was not followed.

Now all is changed; and many who sneered at indomitable Walker, groping in graveyards at dead of night to collect sanitary knowledge, as Vesalius climbed the gibbet to steal anatomy, would gladly inspect any nuisance whatever, and solemnly renounce mandragora for a spell at vital statistics. In most cases this change is due to an improved knowledge; for the doctors are at once a liberal and a progressive race. Still, the sanitary turn in these last days taken an angle so frightfully acute, that we rather fear the golden calf, which is just set up in our scientific Horeb, has contributed somewhat to the revulsion of feeling at this moment exhibited. The electors of medical officers will do well to ponder on this, and to remember that those who spoke and wrote and laboured for sanitary science for its own sake, are the men most fitted to work for it, now that sanitary labours promise to bring grist to the mill.

### ACTS.

*Metropolitan Buildings Act.*—This act comes into operation Jan. 1st, 1856, and extends to all places within the limits of the metropolis, as defined by the "Act for the better Local Management of the Metropolis." It is divided into five parts.

Part I (clauses 6 to 68) relates to the regulation and supervision of buildings. Clause 12 relates to the structure and thickness of walls. These are to be of brick, stone, etc.; to rest on the solid ground, on concrete, or other solid substructure; to be properly bonded and put together with mortar or cement; no part is to overhang any part underneath it; the projection of the bottom and the height of the footing of every wall, on each side of it, is to be at least equal to half the thickness of the wall at its base.

The walls of buildings of the warehouse class are to be thicker than ordinary; cross walls somewhat slighter. Clauses 13 to 22 provide respectively for recesses and openings, timber in external walls, bressummers, parapets to external walls, party walls above roof, chases in party walls, roofs, chimneys and flues, close fires for conveying vapour, tubes accesses and stairs. Clause 23 relates to habitable rooms, which, except when in the roof or underground, are to be at least seven feet in height from floor to ceiling. When in the roof, they must be of the same height throughout, not less than one-half the area of the room. Any person knowingly suffering any room not thus constructed to be inhabited, incurs heavy penalty; any room in which any person passes the night, being deemed inhabited within the meaning of the act. Clause 26 relates to projections, which, amongst other conditions, must be so constructed as to prevent the water therefrom dropping upon, or running over, any public way. Clause 29 provides that every building used as a dwelling-house, unless all the rooms can be lighted and ventilated from a street or alley adjoining, shall have in the rear or on the side thereof an open space, exclusively belonging thereto, of the extent of at least 100 square feet.

*Dwellings for the Labouring Classes.*—By an act passed 14th August, 1855, any number of persons, not fewer than six, may form themselves into a company for providing dwellings for the labouring classes, with or without private or common gardens, or places of recreation; the dwellings being constructed as respects drainage, ventilation, water supply, and necessary conveniences, as may be approved by the General Board of Health, and being open to the inspection of any person appointed by that Board at all reasonable times. In case of insufficient drainage, ventilation, or supply of water, or necessary conveniences, or bad state of repair, the General Board of Health may require the company, under penalty, to make good all deficiencies. The act does not extend to Scotland.

*Dwellings for the Labouring Classes in Scotland.*—By this Act (passed August 14, 1855,) persons associating to erect or improve dwellings, may apply to the sheriff, who will grant his warrant for such erection or improvement, if satisfied as to the ventilation, drainage, sewerage, water supply, security against overcrowding, etc. Clause

19 provides that each separate dwelling shall consist of not less than two, or more than five rooms with fire places. Clause 23 is of considerable importance. It provides that where there shall exist any buildings, which, from faulty construction, or state of delapidation, are unfit for habitation, and the occupation of which in their existing state is attended with risk of injury or disease to the neighbourhood, or which from difficulties as to legal title, may have become a place of harbourage for dissolute and vicious characters, or in any way a nuisance to the neighbourhood, any association desirous of acquiring the same for improvement, repair, or the erection of new dwellings on their site, may apply to the magistrates and town council, who, after inquiry, may require the proprietors, or reputed proprietors of such buildings to rebuild, alter, repair, and drain the same as they may deem necessary for the avoidance of such risk or nuisance in time to come, and to find security that such buildings shall not thenceforward be allowed to afford such harbourage as aforesaid; which not being done, the magistrates and town council may give consent for the association to apply to the Court of Session for authority to acquire [illegible] buildings; and the Court of Session may after [illegible] take steps to ascertain the value of the [illegible] sell them to the association (Clause 26[illegible]use 25), and

*Burial of the Dead in* [illegible]*.*—This Act was passed 14th of August 1855. Clause 8 provides that one of the Secretaries of State [illegible] at any time authorise the inspection of any burial ground to ascertain its state, and whether any regulations that may have been made under other acts have been observed. By clause 9, an act of 15 & 16 Vict. relating to the appropriation of ground for burials nearer than 200 yards to any dwelling-house is repealed; and it is enacted that no ground, not already used or appropriated for burials, shall be used within the distance of 100 yards, without consent from the owner, lessee, or occupier of such house. The Local Board of Health exercises the powers of the Act.

*Burial of the Dead in Scotland.*—By an Act passed 23rd of July, 1855, it is lawful for any two members of the parochial board of any parish in Scotland, or for any ten persons assessed for relief of the poor within such parish, or for any two householders residing within one hundred yards of any existing or proposed burial-ground, to present a petition to the sheriff of the county, setting forth that it is or would be dangerous to health, or offensive to decency; whereupon the sheriff shall advertise a day for inquiry, on which day all parties whom he shall judge to have an interest are to be heard; and should he deem the allegations true, he shall pronounce judgment to that effect, and transmit notice to one of the Secretaries of State for the final order of Her Majesty in Council. Should any petition be dismissed by the sheriff, no other can be presented for five years, except with concurrence of the procurator fiscal. Clause 7 provides, that no Order in Council, unless expressly mentioned, shall extend to burial-grounds of Quakers, or Jews, or the non-parochial burial-grounds of any private person. Clause

21 provides, that one of the Secretaries of State may make regulations in relation to burial-grounds, such as may seem to him proper for the protection of the public health and the maintenance of public decency. Clause 22 exempts burial processions from tolls.

*Diseases Prevention Act.*—This Act, passed August 14, 1855, proposes to substitute more effectual provisions as to the prevention or mitigation of epidemic, endemic, or contagious diseases, than those of the "Nuisances Removal and Diseases Prevention Act, 1848", and the Act for its amendment of 1849.

The local authority to execute the Act is to be the local authority acting in execution of any general Act in force for the time being for the removal of nuisances. Clause 4 provides power of entry for the purposes of the Act. Clause 5, that when any part of England appears to be threatened with, or is affected by any formidable epidemic, endemic, or contagious disease, three or more of the Privy Council (the Lord President of the Council, or one of the Secretaries of State being one), may direct the provisions of this [illegible]ct to be put in force in England, or parts of England, their order [illegible] in force six months, when not otherwise stated, being subj[illegible] to time to revocation or renewal, and being published in the [illegible] Clause 6 provides, that whilst, and so far as any such order is [illegible] in force, the General Board of Health may issue regulations as well as revoke, [illegible]ew, or alter them, as to the speedy interment of the dead, house to house visi[illegible]tation, the dispensing of medicines, medical aid, requisite accommodation; [illegible]these regulations likewise (Clause 7) being published in the *London Gazette.* Clause 8 provides, that the local authority shall attend to the execution of such regulations, appointing and paying medical or other officers, and doing all necessary to mitigate the existing disease. Clause 9 provides prosecution by the local authority for wilful violation or neglect of regulations. Clause 11 provides that Orders in Council may extend to parts and arms of the sea lying within the jurisdiction of the Admiralty; and that the Board of Health for England may issue regulations for cleansing, purifying, ventilating, disinfecting, providing medical aid and accommodation, and preventing disease in vessels, both on parts of the sea and upon inland waters. Clause 12 provides, that when, in compliance with orders of the General Board of Health, any union medical officer shall perform any medical service on board of any vessel, he shall be entitled to charge extra, at the general rate of his allowance for union services, the charges being payable by the captain of the vessel, on behalf of the owners, together with any reasonable expenses for the treatment of the sick. For services rendered on shipboard, any other medical practitioner shall be entitled to charges, and payable in the same way, and extra remuneration for distance, at the rate he is in the habit of receiving from private patients of the class of those attended on shipboard: disputes, where the charges do not exceed £20, to be determined summarily, the justice determining according to the rate of charge within the place, for patients of a like class.

